# MXene Surface Architectonics: Bridging Molecular Design to Multifunctional Applications

**DOI:** 10.3390/molecules30091929

**Published:** 2025-04-26

**Authors:** Wenxuan Huang, Jiale Wang, Wei Lai, Mengdi Guo

**Affiliations:** Hubei Key Laboratory of Energy Storage and Power Battery, School of Optoelectronic Engineering, Hubei University of Automotive Technology, Shiyan 442002, China; 18772453130@163.com (W.H.); wjl2084580201@163.com (J.W.)

**Keywords:** MXene, surface modification, 2D materials, nucleophilic substitution, ligand grafting

## Abstract

This review delves into the surface modification of MXenes, underscoring its pivotal role in improving their diverse physicochemical properties, including tailor MXenes’ electrical conductivity, mechanical strength, and wettability. It outlines various surface modification strategies and principles, highlighting their contributions to performance enhancements across diverse applications, including energy storage and conversion, materials mechanics, electronic devices, biomedical sciences, environmental monitoring, and fire-resistant materials. While significant advancements have been made, the review also identifies challenges and future research directions, emphasizing the continued development of innovative materials, methods, and applications to further expand MXenes’ utility and potential.

## 1. Introduction

Since their groundbreaking discovery by Yuri Gogotsi in 2011 [1], MXenes have rapidly expanded into a diverse family of over 50 members, each exhibiting unique microstructures and stoichiometries [2]. Notable examples include Ti_3_C_2_T_x_ [3], V_2_CT_x_ [4], Mo_2_CT_x_ [5], which have garnered significant attention due to their exceptional properties. MXenes boast a high specific surface area [6]; remarkable mechanical and thermal stability [7]; and a rich surface chemistry [8], coupled electrical conductivity [9], and versatile elemental composition [10]. Compared to other two-dimensional (2D) materials, such as graphene, transition metal dichalcogenides (TMDs), and black phosphorus, MXenes exhibit unparalleled advantages in surface composition, tunability, and property modulation, as demonstrated in Table 1. These attributes have made MXenes indispensable in various fields, including energy storage and conversion [11], desalination [12], electromagnetic interference shielding [13], and biomedical diagnostics [14]. A crucial factor influencing the electrocatalytic and physicochemical properties of MXenes is their surface chemistry [15]. Specifically, the functional groups attached to the MXene surface play a pivotal role in determining their performance. For instance, O-functionalized MXenes exhibit superior capacity in lithium-ion batteries compared to F- and OH-functionalized MXenes. Conversely, MXenes terminated with =O show lower activity in hydrogen evolution reactions due to the strong interaction between hydrogen and the surface [16,17,18]. In contrast, MXenes terminated with non-native functional groups such as -P, -Si, -S, and -Cl often exhibit more positive effects [19,20,21]. However, MXenes typically synthesized by chemical etching methods contain abundant =O, -OH, or -F groups, which provide good hydrophilicity and chemical activity [22]. Despite these advantages, these functional groups can hinder the transport of electrolyte ions, thereby reducing the energy storage potential of MXenes and limiting their further development and application [23,24,25]. A comprehensive understanding of the surface chemistry and reactivity of MXenes is essential for the development of processable functional materials and for paving the way for their application in various fields. Research in this area is ongoing, with scientists working tirelessly to develop new methods for synthesizing MXenes with tailored surface chemistries. By fine-tuning the surface functionalization of MXenes, it is possible to optimize their performance in energy storage, energy conversion, and other applications. This review not only holds promise for advancing functional materials but also lays the foundation for the innovative and sustainable use of MXenes in various fields.

MXenes are commonly synthesized by selectively etching the intermediate A layer from the M_n+1_AX_n_ phase using acids or molten salt, where M represents early transition metals, A signifies group A elements, and X is carbon (or nitrogen), with n ranging from 1 to 3. Alternatively, MXenes can be synthesized directly via chemical vapor deposition. A critical factor in dictating MXene properties is the extent of surface functional group coverage, which largely depends on the chosen synthesis route [30]. For instance, utilizing fluorine-based reagents (typically NH_4_HF_2_ [31], KHF_2_, and NaHF_2_ [32]) during the etching process can result in a high density of -F and =O groups on the MXene surface due to the formation of strong Ti-F and Ti-O bonds. In contrast, adopting fluorine-free methods like molten salt synthesis yields MXenes contaminated primarily with -Cl or -Br impurities, which can be adjusted by post-synthesis treatments like heating or chemical processes to achieve tailored surface functionalities [33,34,35,36]. In addition to manipulating surface functionalities by basic methods, researchers have been exploring innovative ways to modify MXene surfaces. Inserting or grafting organic molecules and polymers, such as magnolol [37], acrylic acid [38], and amine groups [39], onto MXene surfaces enhances their material stability, mechanical strength, and antioxidant properties, respectively. Consequently, understanding the relationship between the molecular configuration, electronic structure, and chemical/electrochemical properties of these surface modifiers is indispensable for designing MXene-based devices tailored for various applications. As the research community continues to uncover new methodologies and properties of MXenes, their potential applications across different sectors are expected to grow exponentially.

**Figure 1:**
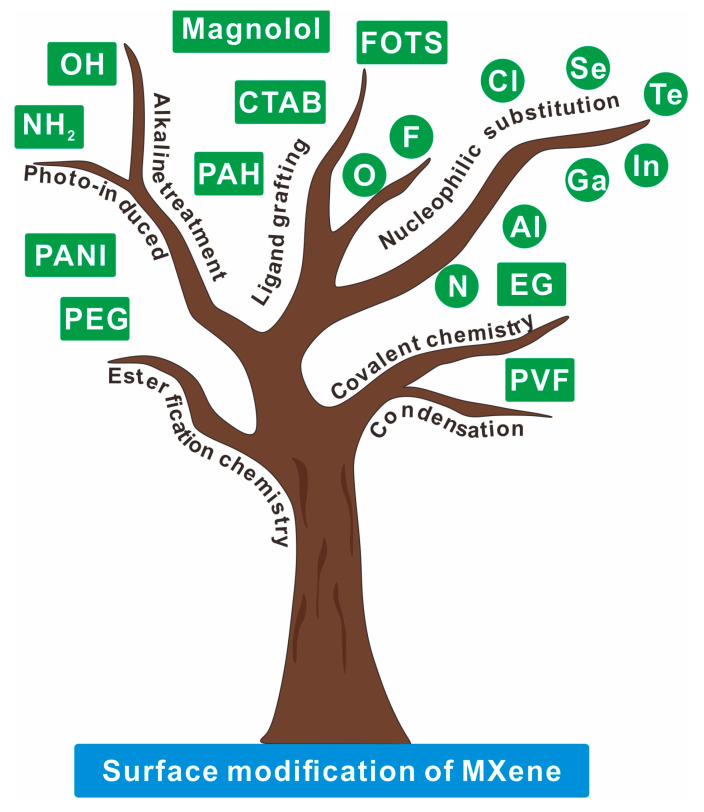
Schematic illustration of the surface modification of MXene.

The surface modification of MXenes is critical due to the instability of exposed metal atoms and uncontrollably embedded ions during synthesis, which lead to oxidative degradation and a sharp decline in activity and physicochemical properties [40,41,42]. Tailoring surface terminal groups (e.g., –F, –OH, –O, –NH) enables the precise modulation of optical (plasmon energy and photoluminescence) and electrical (work function and superconductivity) properties [43,44,45,46,47]. Surface passivation not only enhances stability but also allows the tailored design of functionalized MXenes for diverse applications [48]. Herein, this review systematically elaborates on how diverse MXene synthesis methods dictate surface functional group configurations; highlights their applications in energy storage, structural reinforcement, electronics, biomedicine, and environmental protection through surface modification strategies; summarizes performance parameter enhancements achieved via surface decoration; and ultimately discusses both current challenges and future prospects in surface-engineered MXene development. Initially, this review encapsulates the diverse strategies employed for MXene surface modification, as illustrated in Figure 1, highlighting how they alter surface functional groups and result in improved structure, performance, and stability. The advancements achieved in fields like energy storage, lubricants, and biomedicine through these modifications are also underscored. Moreover, the review delves into the limitations and challenges of current synthesis methods in MXene surface modification, while also presenting future application trends and the developmental significance of MXene surface engineering.

## 2. Approaches for MXenes’ Surface Modification

### 2.1. Synthesis Routes of MXene

**Figure 2:**
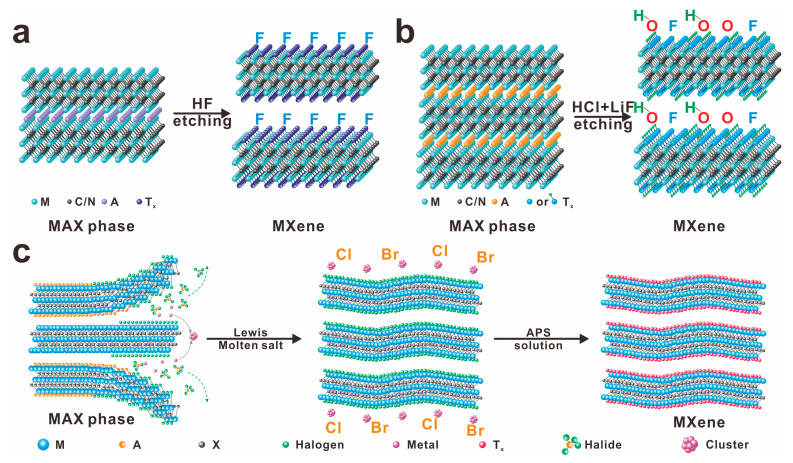
Diagram of MXene synthesis by selective etching MAX phase: (**a**) HF-based acidic etching method, resulting in abundant fluorinated groups (-F); (**b**) fluoride salt based acidic etching method, leading to the distribution of various surface groups including =O, -OH, -F; and (**c**) Lewis molten salt etching method for generation of halogen group (-Cl or Br), reprinted with permission from [52]. Copyright 2024 Wiley.

Almost all MXene materials are derived from the selective etching of the MAX phase, utilizing methods such as hydrofluoric acid etching [49], fluoride salt etching [50], and Lewis acid molten salt etching [51], as depicted in Figure 2. The selection of the etching method is determined by the composition of the A group and the transition metal M in the MAX phase. Acidic etching is effective for phases containing Al and Si atoms, while molten salt etching can be applied to Al, Si, Zn, and Ga. The mild strategy involving the addition of fluoride salts and hydrochloric acid offers gentler reaction conditions and broader acceptance. Regardless of the etching method employed, acidic etching typically results in MXenes with surface functional groups such as -F, =O, and -OH. In specific conditions, such as under electrical bias or through hydrothermal treatment, -Cl groups can also be obtained. In contrast, molten salt etching using ZnCl_2_ yields MXenes with pure Cl-terminated surfaces, and subsequent research has led to the development of Ti_3_C_2_ with Br-terminated surfaces [34,35]. These surface -Cl and -Br groups can undergo exchange reactions with other atoms, forming surface functional groups that can further react with new substituent molecules. This ability to manipulate surface functionalities allows for the production of MXenes with diverse physicochemical properties, expanding their potential applications.

In addition, computational studies reveal MXene surface functionalization (e.g., -F, -O, halides) as a key lever to tailor electronic, catalytic, and mechanical properties. DFT analyses show surface terminations in Ti_2_XT_2_ MXenes reduce NRR limiting potentials (e.g., from −1.24 V to −0.21 V) by altering charge distribution and work functions, with ΔG_*NNH2_ emerging as a predictive descriptor [53]. Functionalization strategies (covalent/noncovalent) enable tunable conductivity, exemplified by Ti_2_C–O_2_’s semiconductor transition or Dirac-cone features in halogenated Ti_3_C_2_ [54]. Surface groups also enhance mechanical adaptability for flexible devices. These insights guide MXene optimization for energy storage, electrocatalysis (NRR, HER), and sensors by linking surface chemistry to performance. Challenges remain in scalable synthesis and experimental validation, urging integrated theory-experiment approaches for next-gen applications.

### 2.2. Surface Modification of MXene

**Figure 3:**
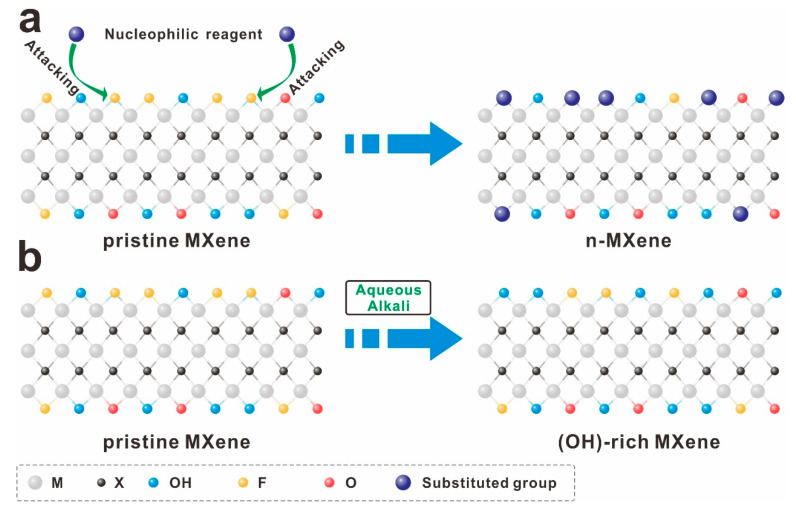
Surface engineering of MXene with nucleophilic substitution reaction (**a**) and alkali treatment (**b**).

Due to the abundance of electron-deficient F-terminated groups on the MXene surface, these groups can be strategically functionalized with electron-withdrawing groups through affinity substitution reactions [55], as depicted in Figure 3a. This approach effectively removes F-terminations and introduces hydroxyl-containing nucleophilic groups, enhancing the pseudocapacitive performance of MXenes [56]. Alternatively, soaking MXenes in basic solutions such as KOH, NaOH, or LiOH can remove F-terminated groups by increasing -OH termini [57,58,59], as illustrated in Figure 3b. However, while this soaking method is straightforward, it exhibits relatively low efficiency in group substitution.

**Figure 4:**
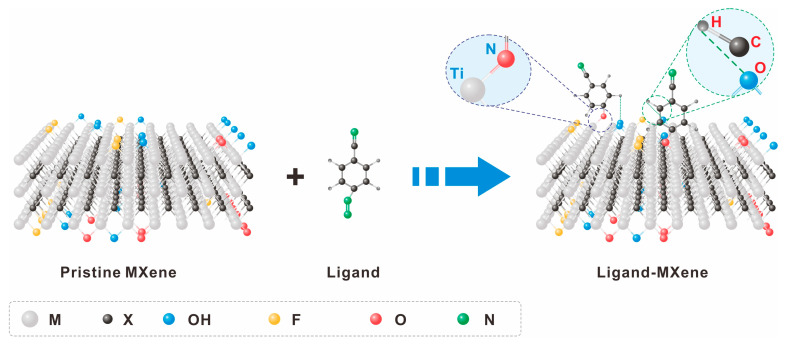
Surface engineering of MXene via ligand grafting. The ligand molecules are grafted onto the surface of MXene via Ti-N bonds, while forming partially unstable hydrogen bonds.

To prevent MXene surface oxidation and enhance its reprocessing performance, various ligand grafting strategies have been employed for MXene surface modification, such as catechol (tailoring the colloidal stability) [60], silane (achieved a 0.0001–2000 ng mL^−1^ with sensitivity of 37.9 µA ng^−1^ mL cm^−2^ per decade for CEA) [61], isocyanate (successful dispersion within a hydrophobic thiourethane matrix) [62], phosphoric acid (over 30 days of storage compare to 2 days of pristine MXene) [63], diazonium (on/off current ratio of 3.56) [64], and amine ligands (324 F g^−1^, twice value of pristine MXene) [65]. These ligands anchor to the MXene surface via hydrogen bonds, electrostatic interactions, and covalent bonds formed between atoms like O, N, and S in the ligand and atoms on the MXene surface, as illustrated in Figure 4. Notably, nitrogen-containing ligands can form more stable and effective covalent bonds with MXene due to the charge transfer effect between N and Ti atoms [66].

**Figure 5:**
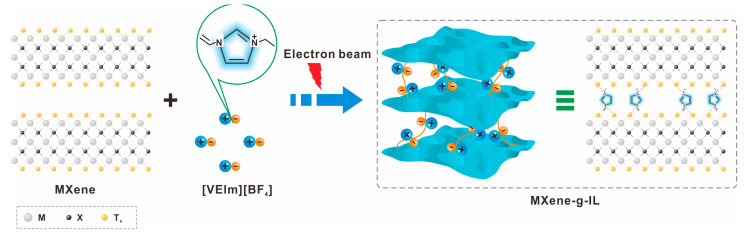
Enhancing the surface properties of MXene through irradiation methods such as lasers and electron beams for improved functionality.

Surface modification of MXenes using external stimuli such as laser and plasma methods has been shown to significantly enhance their performance [67]. A study by Jiang et al. demonstrated the successful grafting of ionic liquid onto Ti_3_C_2_T_x_ interlayers using a simultaneous irradiation method, resulting in a specific capacitance of 160 F g^−1^ and improved structural stability lasting 180 days [68], as shown in Figure 5. Furthermore, one-step plasma treatment was utilized to nitrogen-dope Ti_3_C_2_T_x_, resulting in higher electronic conductivity. This modified MXene was then applied to construct a high-performance Li-S battery [69]. These findings highlight the potential of using external stimuli for surface modification of MXenes to optimize their performance in various applications.

## 3. Applications

### 3.1. Energy Storage and Conversion

**Figure 6:**
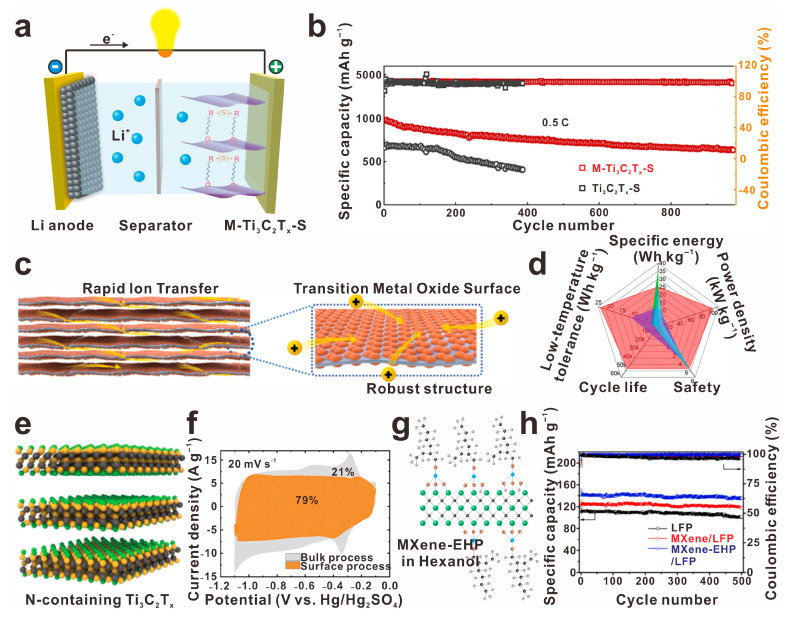
Surface modification of MXene materials holds promise for enhancing energy storage and conversion performance in various applications. (**a**) Schematical illustration of M-Ti_3_C_2_T_x_-S-based Li-S battery and (**b**) the corresponding cycle performance and stability, reprinted with permission from [37]. Copyright 2022 Elsevier. (**c**) Ti_3_C_2_O_y_-derived electrode and (**d**) the electrochemical performance, reprinted with permission from [70]. Copyright 2024 Wiley. (**e**) The atomic structure of N-containing Ti_3_C_2_T_x_ and (**f**) the CV performance of the N–Ti_3_C_2_T_x_-based capacitor, reprinted with permission from [71]. Copyright 2023 Wiley. (**g**,**h**) molecular structure of EHP-modified MXene and the corresponding electrochemical performance of the MXene/EHP-based supercapacitor, reprinted with permission from [74]. Copyright 2024 Elsevier.

Surface-modified MXenes have proven to enhance the conductivity, oxidative stability, and structural stability of various materials. In a study by Zhang and colleagues, Magnolol-modified Ti_3_C_2_T_x_ (M-Ti_3_C_2_T_x_) was designed as a cathode, resulting in an electrode with a high reversible capacity of 7.68 mAh cm^−2^, even when loaded with a high sulfur content, as depicted in Figure 6a,b [37]. This was achieved with a low decay rate of 0.07%, indicating the effectiveness of chemisorption and C-S covalent bond formation in suppressing lithium polysulfide shuttling. Furthermore, Xu et al. introduced a nearly full oxygen-functionalized MXene (Ti_3_C_2_O_y_) through a nucleophilic substitution reaction, leading to the development of electrode host materials with ultrahigh density Ti─O/=O redox-active sites [70], as illustrated in Figure 6c. This resulted in high gravimetric and volumetric capacitance (1082 F g^−1^ in a potential window of 0.85 V), fast charging/discharging rates (tens of seconds, −70 to 60 °C); excellent structural and chemical stability; and improved low-temperature tolerance, power density, cycle life, and safety (Figure 6d). Liu et al. prepared Cl-terminated MXenes (Ti_3_C_2_Cl_x_) and N-terminated MXenes using molten salt etching and nucleophilic substitution reaction methods, respectively [71], as represented in Figure 6e. The N-containing Ti_3_C_2_T_x_ exhibited exceptional rate performance with a unique capacitive-like electrochemical signature and achieved a rate performance of 300 F g^−1^ at 2 V s^−1^ (Figure 6f). Moreover, Tian et al. developed amino-rich (–NH– and –N^+^H–) surface-functionalized Ti_3_C_2_T_x_ (N–Ti_3_C_2_T_x_-200), which facilitated the ion transmission of hydrogen [72]. When incorporated into supercapacitors alongside a copper hexacyanoferrate cathode, the system achieved a wide voltage window (2 V) and high energy density (104.9 Wh L^−1^ at 0.38 kW L^−1^). Additionally, a cyclo-crosslinked polyphosphazene-modified Ti_3_C_2_T_x_ (Ti_3_C_2_/PZS) was synthesized to improve ion transport, accessibility, and oxidation resistance [73]. The resulting supercapacitor exhibited a high pseudocapacitance of 380 F g^−1^. Finally, 2-Ethylhexyl phosphate (EHP) was grafted onto the surface of Ti_3_C_2_T_x_ (Ti_3_C_2_T_x_/EHP) to enhance dispersion stability and compatibility with its counterparts [74], as displayed in Figure 6g. This modification led to the development of Ti_3_C_2_T_x_/EHP/LFP with high specific capacity (150 mAh g^−1^) and cycle stability (136 mAh g^−1^ after 500 cycles at 1 C, Figure 6h), making it a promising material for energy storage applications.

### 3.2. Reinforcing Material Strength

Enhancing the mechanical properties of MXenes is essential for advancing their utilization in various applications such as gels, flexible electronic devices, and additives. Researchers have conducted innovative studies to improve the performance of MXenes through different methods.

**Figure 7:**
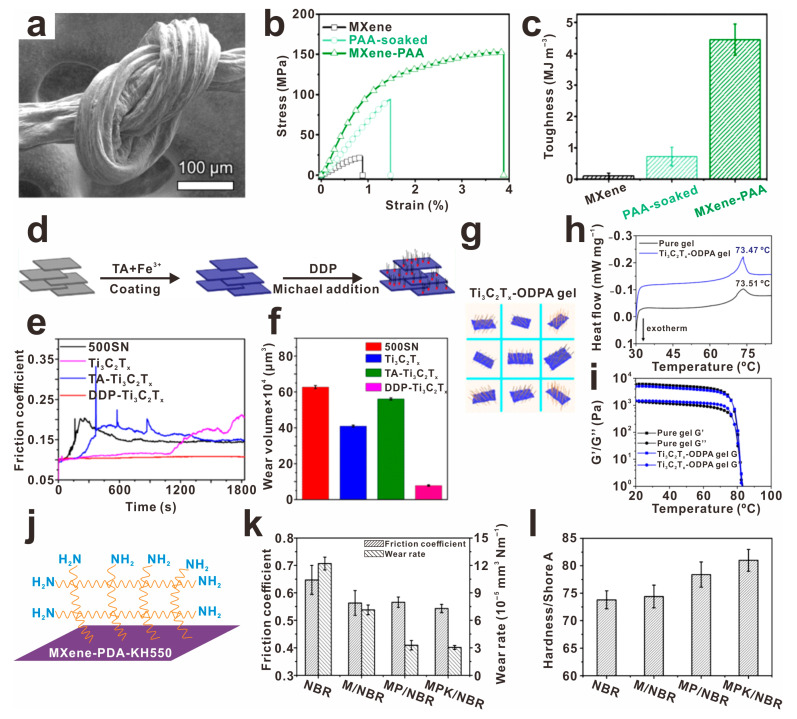
The advancement of composite materials has seen a significant improvement in mechanical properties through surface modification techniques, as follows: Increasing mechanical strength through poly (acrylic acid) grafting on Ti_3_C_2_T_x_, (**a**) the corresponding SEM image, (**b**) stress–strain curves, and (**c**) breaking energy (toughness) values of Ti_3_C_2_T_x_-PAA, reprinted with permission from [38]. Copyright 2024 Elsevier. Improving the anti-wear and friction reduction capabilities in lubricant additives through (**d**) dialkyl dithiophosphate-modified Ti_3_C_2_T_x_ (DDP-Ti_3_C_2_T_x_) and (**g**) octadecylphosphonic acid-modified Ti_3_C_2_T_x_ (Ti_3_C_2_T_x_-ODPA). And the corresponding COF/time curve (**e**) and wear volume losses (**f**) of DDP-Ti_3_C_2_T_x_,, reprinted with permission from [75]. Copyright 2021 American Chemical Society. The corresponding DSC curves (**h**) and evolution of G’ and G″ along with the viscosity (**i**) of Ti_3_C_2_T_x_-ODPA, reprinted with permission from [76]. Copyright 2022 American Chemical Society. (**j**) Enhancing wear-resistance and prominent damping properties of nitrile butadiene rubber by surface-modified Ti_3_C_2_ with polydopamine and amino silane (Ti_3_C_2_-PDA), (**k**) the corresponding COF and wear rate and (**l**) shore A hardness of Ti_3_C_2_-PDA, reprinted with permission from [77]. Copyright 2021 Elsevier.

Firstly, Usman et al. prepared an LC-MXene (MXene-PAA) via the spontaneous polymerization of acrylic acid. Usman et al. have successfully developed an LC-MXene (MXene-PAA) by utilizing a spontaneous polymerization process involving acrylic acid (Figure 7a) [38]. The inclusion of acrylic acid not only helps in preventing oxidative reactions but also aids in the coagulation of extruded MXene-PAA dispersions to form durable fibers. The resulting spinning fiber exhibits an impressive tensile strength of 155 MPa and breaking energy of 4.5 MJ m^−3^, as plotted in Figure 7b,c, respectively. In the quest for more effective lubricants with enhanced friction reduction capabilities and anti-wear properties, Gao et al. introduced a commercial lubricating additive called dialkyl dithiophosphate-modified Ti_3_C_2_T_x_ (DDP-Ti_3_C_2_T_x_) [75], as illustrated in Figure 7d. This additive showcased a low coefficient of friction (~0.11, Figure 7e) and reduced wear volume (~87%, Figure 7f), indicating its potential as a superior lubricative additive. Further advancements in reducing friction coefficients and wear volumes were achieved by Guo et al. through the addition of octadecylphosphonic acid-modified Ti_3_C_2_T_x_ into solvent neutral (SN) supramolecular gel [76], as illustrated in Figure 7g–i. This modification resulted in a significant improvement, with a reduction of 46.32% in friction coefficient and 81.18% in wear volume. Qu et al. introduced surface modifications using polydopamine and amino silane to enhance the properties of Ti_3_C_2_T_x_ (Ti_3_C_2_-PDA) through solution blending and hot pressing methods [77], as illustrated in Figure 7j. The developed Ti_3_C_2_-PDA-KH550 composite demonstrated a remarkable decrease in friction reduction (Figure 7k), smaller wear rate (76%), and increased hardness (Figure 7l), showcasing its potential for various applications needing enhanced mechanical properties.

### 3.3. Electronics

**Figure 8:**
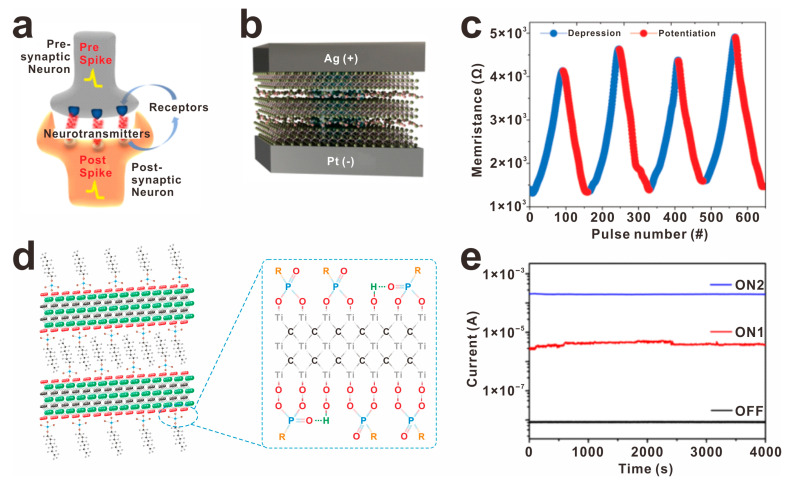
The surface modification of MXene has been shown to significantly enhance the performance of electronic devices, particularly memristors, enhancing the memory window and low-power operation of the Ti_3_C_2_T_x_ memristor via surface modification. (**a**) Schematic diagram of a biological synapse, (**b**) schematic layout of the device as an artificial electronic synapse, and (**c**) potentiation of the MXene-based memristor, reprinted with permission from [78]. Copyright 2023 Wiley. (**d**) Atomic structure of Octylphosphonic acid-modified Ti_3_C_2_T_x_ (OP-Ti_3_C_2_T_x_) and its retention time (**e**), reprinted with permission from [79]. Copyright 2020 American Chemical Society.

The exceptional physicochemical properties of MXene have paved the way for its successful application in memristor devices, particularly in the realm of resistive-switching memory and artificial synapse technology. Researchers, including Mullani et al. and Sun et al., have made significant advancements in this field. Firstly, Mullani et al. developed an O-rich terminated Ti_3_C_2_T_x_-based memristor [78], showcasing low threshold voltages (V_SET_ = 1.33 V and V_RESET_ = −0.94) and high-density memory functionality (>10^5^), as illustrated in Figure 8a–c. Meanwhile, Sun and colleagues also contributed to this progress by introducing an octylphosphonic acid-modified Ti_3_C_2_T_x_ (OP-Ti_3_C_2_T_x_) as an active layer in memristors (Figure 8d) [79], achieving low threshold voltage, stable retention time, distinct resistance states, and a high ON/OFF rate (OFF/ON1/ON2 = 1:10^2.7^:10^4.1^), as depicted in Figure 8e. These surface-engineered architectures not only achieve multilevel data storage but also mimic biological synaptic functions like paired-pulse facilitation and spike-timing-dependent plasticity, bridging the gap between high-density memory and neuromorphic computing within a single device platform.

### 3.4. Biomedicine

**Figure 9:**
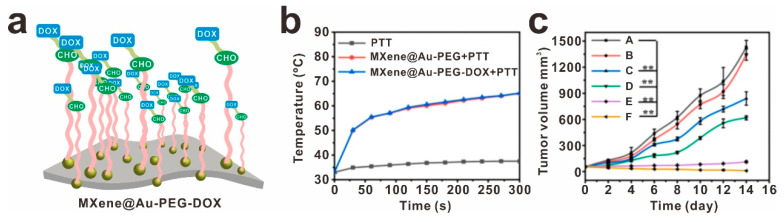
Enhancing drug delivery efficiency is crucial for improving cancer treatment. (**a**) Surface modification of MXene@Au using thiol polyethylene glycol aldehyde chains (SH-PEG-CHO) (MXene@Au-PEG-DOX), (**b**) the temperature curves for tumors, (**c**) and tumor volume growth curves, Control (**A**), PTT (**B**), DOX (**C**), MXene@Au-PEG-DOX (**D**), MXene@Au-PEG +PTT (**E**), and MXene@Au-PEG-DOX + PTT (**F**), reprinted with permission from [80]. ** *p* < 0.05. Copyright 2022 Elsevier.

MXene, known for its high drug-loading capacity, versatile drug release modes, and excellent biocompatibility, has emerged as a promising option for drug delivery applications. In particular, the development of an MXene@Au-PEG drug delivery platform for loading the chemotherapy drug doxorubicin (DOX) has shown great potential [80], as illustrated in Figure 9a. Through in vivo and in vitro testing, this system has exhibited exceptional photothermal stability, biosafety, and histocompatibility. Furthermore, the MXene@Au-PEG-DOX platform has demonstrated the ability to provide synergistic photothermal ablation and chemotherapy effects (Figure 9b,c). In a groundbreaking study by Korupalli et al., the surface of Ti_3_C_2_ was modified with antioxidants (sodium ascorbate and dopamine, DSTC) to enhance its antioxidant capacity [81]. Subsequently, biomolecules with functional groups were loaded onto the surface, leading to the development of CGDSTC NSs. These NSs were further conjugated with enzyme glucose oxidase (GOx) and photosensitizer Ce6, exhibiting impressive photothermal effects with a high conversion efficiency of 45.1% and potent photodynamic properties upon irradiation with 808 and 671 nm lasers. These innovative approaches hold significant promise for the future of drug delivery and therapeutic interventions. Due to the limitations in the application of MXene in the biomedical field, further attempts and expansions of surface modifications on MXene are needed.

### 3.5. Environmental Protection

**Figure 10:**
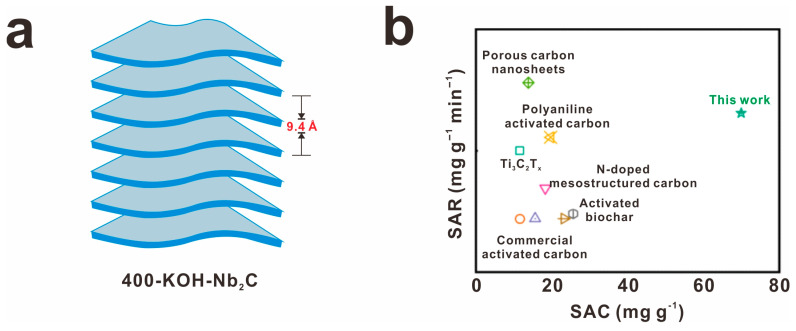
The importance of environmental protection is highlighted in recent studies focusing on the removal of radioactive barium ions and other solute elements from simulated nuclear wastewater. (**a**) 400-KOH-Nb_2_C and (**b**) the corresponding desalination performance compared with others, reprinted with permission from [83]. Copyright 2024 Elsevier.

The treatment of nuclear wastewater is a critical factor in maximizing the potential benefits of nuclear energy. In a recent study by Mu et al., a novel material, known as Alk-Ti_3_C_2_T_x_, was developed with a unique structure featuring wide layer spacing and numerous active adsorption sites [82]. This material demonstrated a significantly enhanced ability to adsorb barium ions, with a capacity of 46.46 mg g^−1^, three times higher than the pristine MXene. The selectivity of Alk-Ti_3_C_2_T_x_ was also found to be exceptional in simulated conditions, highlighting its effectiveness in wastewater treatment. In a similar study, Ashebo and his team successfully synthesized Nb_2_CT_x_ with =O terminations using KOH treatment and thermal annealing [83]. This modification led to a notable increase in salt adsorption capacity (104.2 mg g^−1^ at 1.6 V) and an impressive removal rate of 1.73 mg g^−1^. The enhanced performance of 400-KOH-Nb_2_C can be attributed to the expanded interlayer spaces and higher concentration of =O terminations, which facilitate faster ion transport (Figure 10). These findings underscore the potential of these advanced materials in improving the efficiency and safety of nuclear wastewater treatment processes. In addition, MXene surface modifications have great potential for expanding applications in the adsorption and treatment of VOCs in the air, soil remediation, and protection against light pollution.

### 3.6. Others

**Figure 11:**
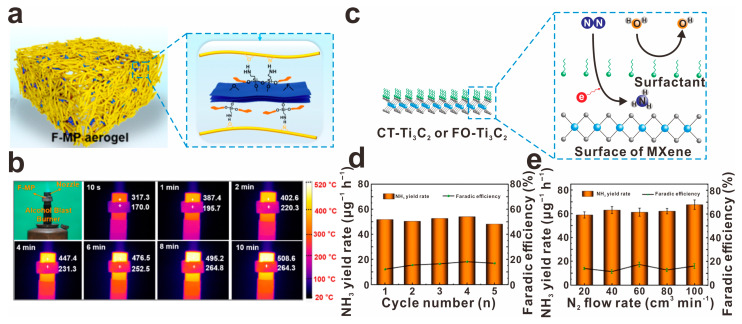
(**a**) Schematical presentation of silane-modified MXene and polybenzazole (F-MP) nanocomposite aerogels, and (**b**) photos and thermographic images of the high temperature thermal insulation test of F-MP aerogel, reprinted with permission from [84]. Copyright 2023 Elsevier. (**c**) Improving nitrogen reduction reaction by cetyltrimethylammonium bromide-modified Ti_3_C_2_T_x_ (CT-Ti_3_C_2_) or FO-Ti_3_C_2_, (**d**) the corresponding NH_3_ yield rate and Faradic efficiency of CT-Ti_3_C_2_ under 5 cycles of testing, and (**e**) NH_3_ yield rate and Faradic efficiency of CT-Ti_3_C_2_ under different N_2_ flow rates, reprinted with permission from [85]. Copyright 2023 Elsevier.

In a groundbreaking development, Wu et al. have successfully created a nanocomposite aerogel by combining silane-modified MXene with polybenzazole [84], as shown in Figure 11a. This innovative aerogel, known as F-MP, boasts exceptional qualities such as low density ranging from 30 to 70 mg cm^−3^, impressive surface hydrophobicity at approximately 141°, and outstanding flame resistance, demonstrated by its structural stability even after a 60 s flame attack, as displayed in Figure 11b. The synergistic effects of the interactions between the 1D polybenzazole and 2D MXene contribute significantly to the unique properties of the F-MP aerogel. Additionally, Wang et al. have adopted a different approach by utilizing hydrophobic molecules like CTAB for the surface modification of MXene [85], as shows in Figure 11c. This modification strategy has proven to enhance both the NH_3_ yield rate, increasing from 37.62 to 54.01 μg h^−1^ ${\mathrm{mg}}_{\mathrm{cat}}^{-1}$ (Figure 11d), and the Faradic efficiency, from 5.5% to 18.1% (Figure 11e). The success of this method can be attributed to the surfactant molecules effectively blocking the entry of water molecules and preventing the concurrent competitive HER, thereby improving the reduction reaction of N_2_. These significant advancements highlight the potential of innovative nanocomposite materials in various applications.

Although the surface modification of MXenes has led to significant success in fields such as energy storage, material reinforcement, and biomedicine, several challenges remain. For instance, there is uncertainty about whether MXenes can be further expanded into other areas, such as antibacterial applications or anti-aging treatments. While surface modifications have indeed improved their performance, the process is often complicated and characterized by unstable conversion rates. Additionally, there are challenges in further modifying MXenes obtained through traditional chemical etching methods. Therefore, efforts should be made to improve synthesis routes, optimize modification strategies, and explore more suitable applications.

## 4. Challenges and Perspectives

Although significant advancements have been achieved in the surface modification and applications of MXenes (as summarized in Table 2), leading to remarkable improvements in properties such as specific capacity, mechanical strength, and adsorption capacity, considerable challenges remain in advancing MXene surface modification toward scalable production and industrial implementation.

### 4.1. Challenges

While many studies have explored the successful insertion of molecular functional groups onto the surface of MXenes, much of the research has predominantly concentrated on Ti_3_C_2_T_x_. This is likely because Ti_3_C_2_T_x_ was one of the earlier MXenes studied and is known for its stable chemical properties. In contrast, other members of the MXene family have not been as extensively researched and possess more active properties. Thus, there is a need to expand surface engineering strategies to include other MXene members in order to enhance their practical applications. By broadening the focus beyond Ti_3_C_2_T_x_, researchers can unlock the full potential of MXenes and harness their unique physicochemical properties for a wide range of applications. In addition, developing new types of MXene and ultimately significantly improving their related properties remains a feasible path (Table 3). It can be clearly seen that the change of components induced a great change in the electrical and hydrophilic properties of MXene, and it even affects its final application field.

While MXene preparation and modification methods typically involve hydrofluoric acid etching and substitution grafting, research on molten salt etching followed by modification is limited. The prevalence of acid etching is attributed to its established nature, whereas the molten salt etching process demands more intricate conditions. Thus, there is a pressing need to enhance and streamline the molten salt etching-based preparation and modification process. This refinement will allow for the creation of MXenes with a wider array of functional group modifications, ultimately expanding the potential applications of these materials in various fields. Emphasizing the development of this alternative method is crucial for advancing MXene technology.

The current research on surface-modified MXenes primarily emphasizes enhancing energy storage and mechanical properties, overlooking their potential in electronics, biomedicine, and environmental remediation. To advance the field, it is crucial to broaden the scope of applications and delve into uncharted territories. Future investigations should prioritize exploring new possibilities stemming from the altered characteristics of MXenes, paving the way for innovative advancements beyond traditional focus areas.

### 4.2. Perspectives

Surface modification plays a crucial role in enhancing the overall properties of MXenes, such as their electrical conductivity, mechanical strength, and hydrophilicity/hydrophobicity. This comprehensive review examines the various strategies and principles employed in the surface modification of MXenes and highlights the resulting improvements in performance across a range of applications including energy storage, materials mechanics, electronics, biomedicine, environmental monitoring, and fire-resistant materials. Furthermore, the review delves into the existing challenges and future research directions in MXene surface modification, emphasizing the need to innovate new materials, techniques, and applications to further advance the field. Ultimately, the surface modification of MXenes shows great promise in revolutionizing various industries with its enhanced properties.

## Figures and Tables

**Table 1:** Surface property of MXenes compared with other 2D materials.

Character	MXenes [26]	Graphene [27]	TMDs [28]	Black Phosphorous [29]
Surface component	Transition metal and carbon/nitrogen layer; the surface is rich in functional groups	Cellular sp^2^ carbon network, surface inert	Layered structure of transition metals with chalcogenide elements	The folded phosphorus layer, the surface of the lone pair electron enrichment
Functional group	(Adjustable) =O, -OH, -F, and -Cl	Need manual introduction: -COOH, -OH, -NH_2_	Natural sulfur vacancies (S-vacancies) can support metal nanoparticles	Oxidation to form P-O/P=O group
Chemical stability	Easy oxidation in air (dependent on functional groups and metal composition)	High (inert surface corrosion resistance)	High (stable under non-extreme conditions)	Very low (inert atmosphere required)
Surface charge	Adjustable (pH-dependent Zeta potential)	Hydrophobic, negatively charged (after oxidation)	Negative charge	Amphoteric (pH responsiveness)
Functionalization strategy	Direct grafting (e.g., silane, polymer), ion intercalation	Covalent bonds (e.g., acylation), non-covalent bonds (π-π packing)	Sulfur vacancy modification, edge doping (Co, Ni, etc.)	Oxidative passivation, polymer encapsulation
Influence	Functional groups regulate electrical conductivity, hydrophilicity, and ionic diffusion rate	Functionalization reduces conductivity but increases dispersion	Sulfur vacancy improves catalytic activity and doping optimizes electronic structure	Oxidation results in a change in band gap and a decrease in carrier mobility

**Table 2:** Comparisons of performance before and after surface modifications of MXenes.

Applications	MXenes	Performance		**Reference**
		Before	After	
Li-S battery	M-Ti_3_C_2_T_x_	815 mAh g^−1^ (capacitance)	1034.5 mAh g^−1^	[37]
Capacitor	Ti_3_C_2_O_y_	323 C g^−1^ (capacitance)	1161 C g^−1^	[70]
Capacitor	N-containing Ti_3_C_2_T_x_	83 mAh g^−1^ (capacitance)	120 mAh g^−1^	[71]
Supercapacitor	N-Ti_3_C_2_T_x_	628.3 C cm^−3^ (capacitance)	936 C cm^−3^	[72]
Supercapacitor	Ti_3_C_2_/PZS	300 F g^−1^ (capacitance)	380 F g^−1^	[73]
Li ion battery	Ti_3_C_2_T_x_/EHP/LFP	118 mAh g^−1^ (capacitance)	150 mAh g^−1^	[74]
Mechanical materials	MXene-PAA	30 MPa (tensile strength)	155 MPa	[38]
Lubricant additives	DDP-Ti_3_C_2_T_x_	0.24 (coefficient of friction)	0.11	[75]
Lubricant additives	Ti_3_C_2_T_x_-ODPA	0.1 (coefficient of friction)	0.046	[76]
Nitrile butadiene rubber composites	Ti_3_C_2_-PDA	0.647 (coefficients of friction)	0.543	[77]
Memristor	Ti_3_C_2_O_x_	10^4^ (ON/OFF ratios)	10^5^	[78]
Memristor	OP-Ti_3_C_2_T_x_	10^2.7^ (ON/OFF ratios)	10^4.1^	[79]
Biomedicine	MXene@Au-PEG-DOX	1350 mm^3^ (tumor volume)	75 mm^3^	[80]
Biomedicine	CGDSTC NSs	-(photothermal conversion of efficiency)	45.2%	[81]
Wastewater treatment	Alk-Ti_3_C_2_T_x_	11.98 mg g^−1^ (Ba^2+^ adsorption)	46.46 mg g^−1^	[82]
Capacitive deionization	400-KOH-Nb_2_C	33.5 mg g^−1^ (salt absorption capacity)	104.2 mg g^−1^	[83]
Aerogel	F-MP	~500 °C (surface temperature)	~264.3 °C	[84]
Reduction reaction of N_2_	CT-TiC_2_	5.5% (Faradic efficiency)	18.10%	[85]

**Table 3:** Influence of element composition on MXene properties.

Component of MXene	Conductivity	Hydrophilicity	Functional Group	Applications	Reference
Mo_2_CT_x_	0.303–4.35	109.3	=O, -OH	Electrocatalytic hydrogen evolution	[86,87]
Ti_3_C_2_T_x_	~500–20,000	90–106.99	=O, -OH, -F	Supercapacitors, electromagnetic shielding	[88,89]
Nb_2_CT_x_	0.0145–0.0923	30–36	=O, -OH	Lithium sulfur battery, photothermal treatment	[90,91]
Ti_2_CT_x_	1.63 × 10^−8^–0.3	45–65	=O, -F	Transparent conducting thin film	[92,93]
V_2_CT_x_	1560	33.6–101.9	=O, -OH, -Cl	Magnetic sensor	[94,95]
Ti_3_CNT_x_	0.128–909	0–25.1	F, -OH, =O	Wear-resistant coating, composite material	[96,97]

## Data Availability

The data that support the findings of this study are available from the corresponding author upon reasonable request.
